# The delicate dance of debriefing: exploring how behavioural marker systems influence the socio-emotional dynamics of simulation practice

**DOI:** 10.1186/s41077-026-00411-4

**Published:** 2026-02-06

**Authors:** Victoria Ruth Tallentire, Scott McColgan-Smith, Fiona Stewart, Seonaid McIntyre, Samantha Eve Smith

**Affiliations:** 1 Medical Education Directorate, NHS Lothian, Edinburgh, Scotland; 2 Medical Directorate, NHS Education for Scotland, Edinburgh, Scotland; 3 Scottish Centre for Simulation and Clinical Human Factors, NHS Forth Valley, Larbert, Scotland; 4 Pharmacy Directorate, NHS Education for Scotland, Glasgow, Scotland; 5 Pharmacy Directorate, NHS Lothian, Edinburgh, Scotland; 6 Centre for Medical Education, School of Medicine, University of Dundee, Dundee, Scotland

**Keywords:** Simulation-based education, Debriefing, Feedback literacy, Educational alliance, Behavioural marker systems, Pharmacists’ behavioural skills, Professional identity formation, Socio-emotional learning, Peer feedback

## Abstract

**Background:**

Debriefing is central to simulation-based education and has been described as both a form of feedback and a broader relational process. While behavioural marker systems (BMS) to assess behavioural (non-technical) skills have been developed to enhance feedback transparency, little is known about how their use shapes the emotional and social dynamics of debriefing. Drawing on feedback literacy and educational alliance frameworks, this study explored how behavioural marker systems influence the emotional and social aspects of debriefing within healthcare simulation.

**Methods:**

This constructivist study involved trainee pharmacists and facilitators participating in a national simulation program in Scotland. Participants used the Pharmacists’ Behavioural Skills BMS, then reflected within online semi-structured interviews. Transcripts were analysed using template analysis. Initial coding was informed by feedback literacy and educational alliance frameworks and refined iteratively through team discussion. The metaphor of dance was used to capture nuance and provide relatable examples of the relational and affective dimensions of practice.

**Results:**

Ten trainee pharmacists and six facilitators participated. Five interrelated themes were identified: *emotional vulnerability (the first tentative steps); clarity of expectations (trusting the choreography); identity work (the dancer in the mirror); peer critique (dancing in sync); agency and ownership (leading the dance).* The behavioural marker system supported learning by depersonalising critique, legitimising peer feedback and enhancing trust through transparency. It also affirmed professional identity and fostered learner agency by making feedback specific and actionable. However, risks were identified when the tool was applied inconsistently, perceived as inauthentic or used in ways that emphasised negative descriptors.

**Conclusions:**

Within simulation, behavioural marker systems are not sterile scaffolds but dynamic social instruments that shape the relational and emotional conditions of debriefing. When applied sensitively, they can depersonalise feedback and strengthen the educational alliance, supporting learners to take ownership of their development. Used rigidly or inconsistently, they risk damaging trust and fragile professional identities. Facilitators should introduce such tools as developmental guides, explicitly aligning them to professional practice and balancing structure with responsiveness to learners’ emotional and relational needs.

**Supplementary Information:**

The online version contains supplementary material available at 10.1186/s41077-026-00411-4.

## Background

The role of feedback within simulation debriefing is an emotive and contested topic amongst simulation educators and academics. While some view debriefing itself as a specialised form of feedback, others emphasise its broader scope. Eppich and Cheng (2015) highlight the broader reflective and socio-emotional functions of debriefing, describing it as “*a conversation between simulation participants and educator(s)*,* [whereas] feedback is the specific information about an observed performance compared with a standard. Effective debriefings can provide a forum for feedback that is essential for performance improvement and deliberate practice that promotes expertise*” [1]. This highlights a central tension: feedback is a critical component of debriefing, yet debriefing also relies on relational and emotional dynamics that extend beyond performance critique.

Emotions are pervasive in simulation-based education (SBE). Learners have described SBE as a “roller-coaster” of anxiety, confusion, relief, pride and hope, with these fluctuations shaping both performance and reflection [2, 3]. Reviews show that emotions influence attention, working memory, decision-making and motivation during SBE, but in complex ways that defy simple categorisation as positive or negative [4, 5]. Educators are encouraged to recognise emotions, assess their impact, and employ strategies to convert potentially disruptive arousal into productive activation [6]. Importantly, emotions in SBE are not experienced in isolation but are intertwined with social structures such as hierarchy, credibility and peer norms, which can amplify or attenuate their effects [7].

Debriefing is a social activity and, as in any feedback moment, learners assess the credibility and trustworthiness of the source before engaging with feedback [8]. Peer presence and peer critique can offer support and constructive comparison, yet may also heighten anxiety or shame if hierarchies are salient or feedback is poorly framed [9, 10]. Facilitators play a pivotal role by attuning to affective cues, “naming and normalising” emotions, and linking affective experiences to clinical reasoning [11]. Thus, both emotion and social context are inseparable from the learning value of debriefing, shaping whether feedback is explored, defended against or dismissed.

Behavioural marker systems (BMS) illustrate how these socio-emotional dynamics intersect with structured feedback. BMS provide predefined categories and exemplar anchors to guide feedback on behavioural skills (also referred to as non-technical skills) such as communication, leadership and teamwork [12]. When introduced into debriefing, they do more than structure content, they influence whose behaviour is noticed, how performance is labelled and how judgements are made visible to peers and facilitators. Widely used systems such as ANTS, NOTSS and Medi-StuNTS have been shown to reduce ambiguity, promote fairness and make critique more acceptable [13–15]. However, the very features that make BMS attractive - explicit criteria, ratings and standardised language - may also amplify learners’ sense of being judged, reinforce existing hierarchies or narrow the focus of discussion if they are used without sensitivity to emotional and relational dynamics. To date, research on BMS has largely examined psychometric properties, implementation and outcomes, with far less attention to how learners *experience* BMS-mediated feedback within debriefing. Despite their growing use in SBE, little is known about how emotional and relational factors influence learners’ engagement with feedback when it is framed through a BMS. A better understanding of this issue could lead to recommendations for using BMS within simulation practice in ways that support, rather than inadvertently undermine, the learning potential of debriefing.

### Theoretical framework

This study draws on two complementary theoretical frameworks: feedback literacy and the educational alliance. Feedback literacy refers to the learner capacities required to understand, engage with and use feedback productively, encompassing appreciation of feedback, evaluative judgment and management of affect [16]. The educational alliance frames feedback as a relational practice grounded in credibility, caring and collaboration between learner and educator [17, 18]. Together, these perspectives emphasise that feedback in SBE is not merely an exchange of information but a relational and emotional process in which trust, perceived fairness and affective safety shape whether, and how, learners take up feedback.

### Impact of behavioural marker systems

When a BMS is introduced into debriefing, it intervenes directly in these processes. By making performance criteria explicit and visible to peers and facilitators, BMS have the potential to enhance feedback literacy (for example, by supporting evaluative judgement and clarifying expectations), and to strengthen the educational alliance (for example, by signalling fairness and transparency). At the same time, the structured ratings and language of BMS may threaten affective safety, heighten a sense of being judged or undermine trust. In other words, BMS are not neutral tools: they may either support or constrain the development and exercise of feedback literacy and the educational alliance within debriefing. Existing work on BMS leaves a gap in understanding how their use shapes the emotional and social dynamics of feedback. It is this gap, at the intersection of BMS, feedback literacy and the educational alliance, that the present study seeks to address. Examining how these dynamics play out in the context of BMS use offers an opportunity to advance understanding of feedback in simulation, and to support facilitators in using such tools with greater nuance and intentionality.

### Research question

Building on existing work utilising the concepts of feedback literacy and the educational alliance, this study aims to address the gap in the literature by asking:

*How does use of a behavioural marker system influence the emotional and social aspects of debriefing within healthcare simulation?*

## Methods

### Ethics

Ethical approval for this study was granted by the NHS Education for Scotland Research Ethics Committee (approval numbers NES/Res/13/20/Ph and NES/Res/30/22/Pharm). All study participants were volunteers and received information about the study before consenting.

### Context

This constructivist study engaged trainee pharmacists and facilitators involved in a simulation program in Scotland. Following completion of their General Pharmaceutical Council accredited qualification (Master of Pharmacy; MPharm), trainee pharmacists in Scotland complete a foundation training year. During the year, designed as preparation for independent practice, the trainees undertake supervised practical experience in clinical settings such as community pharmacies, hospitals or general practice. Additionally, NHS Education for Scotland (responsible for the training of postgraduate pharmacists in Scotland) offers trainee pharmacists a national simulation-based program designed to expose learners to a variety of complex scenarios with debriefing by pharmacist facilitators. Of 219 trainee pharmacists across Scotland, 185 participated in the simulation program run between January and March 2025.

During the simulation sessions, the Pharmacists’ Behavioural Skills (PhaBS) marker system was utilised [19]. PhaBS is a structured BMS designed to enhance feedback on the behavioural skills of trainee pharmacists, tailored specifically to the relevant professional tasks and workplace contexts. Examples of the tri-hierarchical structure (category, element, behaviour) are shown in Table 1.

**Table 1:** Examples of the categories, elements and exemplar behaviours within PhaBS [19]

Category	Element	Positive behaviour	Negative behaviour
Decision-making and prioritisation	Prioritising	Deals with interruptions effectively and professionally	Allows distractions to divert attention from more important tasks
Self-awareness	Speaking up	Challenges colleagues’ decision-making when a threat to patient safety is observed	Fails to communicate urgency or address a patient safety issues

PhaBS was emailed to all participants and facilitators several days before each session, to allow familiarisation prior to the simulation. Before commencement of each session, PhaBS was introduced to all facilitators in a briefing session, where its purpose and content were discussed, with the opportunity to trial its use and ask questions. Trainees were introduced to PhaBS during a separate briefing session prior to the simulation, and both groups were encouraged to provide feedback during the debriefing phase using the examples and language provided by PhaBS. Printed copies of PhaBS were available during the simulation sessions to provide a framework for feedback, with space for notetaking. The sessions were designed such that all participants should have received roughly equivalent exposure to BMS-anchored feedback across the sessions.

### Participant recruitment

Trainee pharmacists attending the national simulation program were recruited to this study on a volunteer basis. Author SMS was the primary organiser of the program and was present at all simulation sessions. Following completion of the program, all trainee pharmacists and facilitators were contacted by email. Those who responded were provided with further information relating to the purpose of the study and the interview requirements. Utilising the concept of information power, study recruitment ceased when the sample size of each group was deemed to adequately address the research question, based on (i) the study aim (narrow and focussed), (ii) specificity of the sample (dense sample who all had direct experience relevant to the research question), (iii) the theoretical grounding of the study (strong and explicit use of theory throughout the research process), (iv) the quality of the interview data (experienced interviewer with articulate participants providing rich responses), and (v) the method of analysis (thematic cross-case comparison) [20]. Other than the method of analysis (broad as opposed to in-depth and case-orientated), the narrow aim, specific sample, strong theory and rich dialogue justified a modest sample size.

### Data collection

An interview guide based on the constructs of feedback literacy and educational alliance was developed collaboratively by all authors (see Appendix 1). Given the wide geographical distribution of participants across Scotland, author VT conducted all interviews online via Microsoft Teams between February and May 2025. During interviews, emphasis was placed on conversation and stories that helped to elucidate the social and emotional aspects of the simulation experience. As such, they were semi-structured in nature and approached from a standpoint of curiosity. Interviews were audio-recorded, transcribed verbatim using Microsoft Teams, and checked for accuracy against the audio file immediately after each interview by VT.

### Data analysis

Data were analysed using template analysis, a form of thematic analysis that emphasises the use of a coding template to organise and interpret qualitative data [21, 22]. Unlike purely inductive approaches, template analysis allows researchers to start with an a priori set of codes, sometimes derived from existing theory, while remaining flexible to modify, add, or delete codes as analysis progresses. The template evolves iteratively through cycles of reading, coding, and refinement, and is applied systematically across the dataset. We therefore elected to use the sensitising theories to form the initial coding template, allowing us to explore data through the lenses of existing constructs (feedback literacy, educational alliance), while still allowing new, data-driven insights to be included.

In this study, the initial coding template was developed by VT based on the key elements of feedback literacy and educational alliance. Once coded into the initial template, areas of overlap, disparity and friction (both within and between the two participant groups) were explored further. Through a process of discussion, negotiation and comparison, the research team collaboratively refined the initial coding template into more nuanced themes that resonated with the data and highlighted consistency or disparity between the two participant groups. During the analytical process, the metaphor of dance featured repeatedly in team discussions: its dynamic, relational nature, shaped by trust within a structured yet improvised context, resonated with the group’s experience of debriefing. As such, the metaphor was formally incorporated into the results to capture the relational and affective dimensions of practice and help readers to see familiar processes differently [23].

### Reflexivity

SMS, FS and SM are academic pharmacists with educational leadership roles and substantial experience of pharmacy education. SES is a general practitioner and educational researcher with a strong qualitative research background. VT is a hospital-based physician with a national simulation role and significant research experience. Together this team have undertaken a program of work on simulation within pharmacy education, including how it might support behavioural (non-technical) skill development, contribute to professional identity formation and enhance tolerance of ambiguity [24–26]. The team also developed and validated the PhaBS marker system and are therefore invested in demonstrating its utility and understanding its limitations [19, 27]. This research journey, along with other work focussed on identity development [28] has undoubtedly influenced this study in numerous ways, from preconceptions and expectations in relation to the research question through to research team relationships and dynamics.

The research team brought particular professional and relational positions to this study that may have shaped participants’ engagement. The first author is a clinician-educator with extensive experience in SBE and the use of BMS, but is not a pharmacist. This ‘insider-outsider’ position may have influenced how trainees and facilitators interpreted the purpose of the study: some may have felt more comfortable sharing critical views of PhaBS with someone outside pharmacy, whereas others may have perceived VT as aligned with institutional simulation practices and therefore moderated their critique. To mitigate this, we emphasised that participation was voluntary, that data would be anonymised, and that contributions would not affect training or assessment outcomes. In terms of methodological reflexivity, we chose individual semi-structured interviews rather than focus groups to enable participants to speak more freely about emotions in debriefing. This likely enhanced the quality and depth of dialogue, but reduced opportunities to observe how participants co-constructed accounts of debriefing with peers. Our thematic, cross-case analysis prioritised identifying patterns over developing very detailed idiographic case accounts. This strategy increased analytic breadth but means that some of the fine-grained complexity within individual narratives may not be fully represented. Throughout analysis, we periodically revisited our assumptions about information power and considered whether additional recruitment was needed; we judged that the existing dataset provided sufficient variation to address the research question, while acknowledging that perspectives from other professions and settings remain an important area for future work.

## Results

Of the 180 trainees and 41 faculty who consented to be contacted after the simulation session, a total of 21 participants agreed to be interviewed. Recruitment ceased after 16 interviews in line with the team’s judgements relating to information power. Demographic information relating to the 16 participants is shown in Table 2. Interviews lasted between 21 and 43 min. Gender identity split was reflective of the overall cohorts of trainee pharmacists in Scotland and facilitators in the simulation program.

**Table 2:** Demographics of participants

	Trainee pharmacists	Facilitators
Gender identity (described in response to an open question)		
Female	9	6
Male	1	0
Age range	22 to 31 years	35 to 52 years
Main location of training / practice:		
Community	7	7
Hospital	2	2
General practice	0	3
Split between community and hospital	2	0
Total	10	6

We drew five main themes from the data. To frame the relational and emotional dynamics underpinning debriefing, we have used the metaphor of dance. Dance captures the essence of debriefing as a process that is simultaneously structured and improvised, requiring coordination, sensitivity, and trust between partners. This metaphor also highlights the risks of exposure and missteps, alongside the potential for confidence growth and shared achievement. ‘TP’ denotes quotes from trainee pharmacists and ‘F’ denotes quotes from facilitators.

### Emotional vulnerability: the first tentative steps

Like stepping onto a dance floor for the first time, debriefing exposes learners to the gaze of others - feeling safe is essential to avoid the risk of missteps and humiliation overwhelming the potential for growth. Learners feel safe when an educator signals investment in the learner and engages in dialogue rather than one-way judgment. Both trainees and facilitators emphasised that simulation feedback carries emotional risk. Trainees described feeling “*slightly daunted… are you going to be judged harshly by your peers or not?*” (TP1). Facilitators recognised this risk and admitted their own hesitancy: *“We’re almost fearful of disrupting that psychological safety by any sort of perceived criticism or negative feedback”* (F3).

However, by grounding critique in predefined categories and observable behaviours, use of a BMS depersonalised feedback and reduced the risk of humiliation: *“We had the tool*,* it was more structured… instead of everyone repeating the same things*,* and without the tool it wasn’t very safe to go beyond that”* (TP6). PhaBS provided clarity of expectations, reducing reliance on personal impressions and opinions. Provision of observable behaviours made critique less subjective, more concrete and seemingly more acceptable to trainees: *“I like the way it was laid out like that and that there were specific examples”* (TP3). The positivity was tinged with caution as there was concern that without careful facilitation, the BMS might risk inhibiting deeper exploration or lead to superficial, checklist-style feedback. In dance terms, PhaBS offered a reassuring basic step, but without sensitive partnering could leave learners marking time rather than moving into more expressive choreography.

### Clarity of expectations: trusting the choreography

In dance, trust builds when partners know the steps and can rely on both the choreography and one another. Similarly, clear expectations and the credibility offered by a BMS enabled trainees and facilitators to engage in feedback with greater confidence. Trust was strongly linked to the transparency and consistency provided by the BMS. Trainees valued having clear expectations, with the basis of judgements visible: “*It was just kind of something to go off… what kind of things are we looking for? What are we critiquing? What are we looking for in the positive and the negative behaviours?”* (TP3). Facilitators also saw the tool as building credibility: *“Having the tool in front of you is good even as a conversation starter… it makes it clear what we’re looking for”* (F1). It also provided facilitators with a way of encouraging trainees to trust in their own judgements: “*They do look to you as the facilitator as the person who has all the answers… but I try to put it back on them to build trust in their own judgments”* (F4).

However, both groups highlighted risks when the system was not consistently applied. One trainee noted inequity: *“I went first*,* so I didn’t get the benefit of that feedback session that other people got… they had the advantage of going in and using the points on the marker system”* (TP3). There was also concern about authenticity, with trainees struggling to see how some categories applied in practice. Thus, while both groups linked trust to clarity and fairness, trainees focused on inequity of access and clinical authenticity of categories within the BMS, and such doubts risked eroding trust in both the tool and the facilitator. In dance terms, uneven or ill-fitting choreography meant some partners felt out of step from the start, undermining their confidence in both the steps and the choreographer.

### Identity work: the dancer in the mirror

The metaphor of the dancer in the mirror captures how feedback through the BMS invited trainees to look at themselves - to see their professional performance reflected back, sometimes with pride, sometimes with discomfort - as they shaped and reshaped their emerging professional identities. The reflections both affirmed progress and, at times, threatening fragile identities. Positive feedback fostered pride and confidence: *“Everyone was really happy with what I did*,* which also made me kind of proud of myself*,* even though I was really nervous inside”* (TP8). Professional identity development was reinforced through use of the BMS providing clear recognition of progress and competence. Furthermore, behavioural descriptors could affirm learners’ sense of progression toward explicit professional norms, thereby strengthening identity alignment with the profession.

Yet negative experiences could be devastating and deeply personal: *“The feedback was a bit too negative… I came away thinking ‘oh my gosh*,* I’m a disaster’”* (TP3). Facilitators recognised this fragility and contextualised it as a cultural issue in pharmacy: *“Pharmacists by nature are perfectionists… suddenly being told they didn’t do something 100% doesn’t land well with them”* (F2). However, the inclusion of negative behavioural descriptors within the BMS risked learners repeatedly receiving feedback framed in deficit terms. Such feedback could threaten their self-concept and undermine their fragile professional identities: *“If they perceive the feedback as annihilating rather than constructive*,* it can really damage their confidence.”* (F3) In dance terms, the mirror intended to refine technique and promote uniformity could instead result in fixation on flaws, reflecting back an image that feels more wounding than helpful.

### Peer critique: dancing in sync

Like a partnered dance, peer critique requires balance and synchronisation. When steps align, the exchange can feel supportive and graceful, but when missteps occur, the risk of treading on toes exposes the social vulnerability inherent in giving and receiving critique among peers. The social risk was recognised by both trainees and facilitators: *“People are a bit reluctant to say negative things about their colleagues”* (TP7), while facilitators observed the same avoidance: *“They don’t want to criticise each other… if they do try*,* it’s kind of fluffed around*,* not directly given”* (F5). At the same time, both groups recognised the potential of a BMS to legitimise peer critique: *“The trainees were asked to try and use the tool when they were watching their peers… you could see that was them trying to put across a different way without directly saying it”* (F6). Through anchoring critique in observable standards, use of the BMS made comments feel more objective: “*It was good to prompt discussion. Without it*,* it can be hard to bring things up without sounding too critical*” (TP10).

However, despite the sugar coating provided by the BMS, peer feedback remained a bitter pill to swallow. Critical peer feedback remained emotionally charged, and use of a BMS did not fully negate the social risk of judging friends or classmates: “*When we were in the debrief session afterwards… there’s a lot more things on the positive side compared to what could have went better. I feel like everyone was holding back*,* because obviously we’re all colleagues… we don’t want to make anyone else feel like they’re doing a bad job*” (TP6). Such reluctance to provide critique to peers to maintain social harmony was, at times, in tension with trainees’ desires for constructive feedback: *“Any feedback that I had that was more critical or negative actually was from me. Other people were maybe not so good at picking up on that”* (TP7). In dance terms, the BMS offered a shared rhythm to guide partners, but many still chose cautious, half-steps rather than risk a misstep that might damage the harmony of the group.

### Agency and ownership: leading the dance

A dance comes alive when a partner confidently takes the lead, shaping the direction and flow. In the same way, feedback fostered agency when trainees moved from passive recipients to active co-constructors of learning and co-ownership of goals and strategies for improvement. The BMS was seen as a personal tool for future self-directed reflection which made the debriefing conversation more concrete and actionable: *“I like the way it was laid out like that and that there were specific examples… we talked through some of the specific points on the form.”* (TP3).

Facilitators were aware of their role in enhancing or constraining agency, and how use of a BMS may risk passivity or disengagement: *“If the facilitator dominates*,* then it takes away from the trainees’ opportunity to own the discussion and their learning”* (F1). There was a sense from both trainees and facilitators that having a written record of the learning points and strategies for improvement was likely to help learners to reflect further and act on the output: *“When it’s written down*,* it probably makes it more impactful than just talking about it”* (F1). In dance terms, the BMS provided a score to follow, but agency emerged when trainees used it to lead the choreography rather than simply be guided through the steps.

### Summary

Table 3 summarises the results and implications for simulation practice, framed in terms of how BMS might be utilised to promote learner interpretation of feedback as credible, consistent and developmental. 

**Table 3:** The potential contributions and risks of BMS, and implications for practice

	Potential contributions of BMS	Risks of BMS utilisation	Implications for simulation practice
*Emotional vulnerability: The first tentative steps*	By grounding critique in predefined categories and observable behaviours, BMS can depersonalise feedback and reduce the risk of humiliation.	If applied rigidly, BMS may heighten the sense of being scored or judged, increasing performance anxiety and inhibiting learning.	BMS can be used to normalise error and emphasise behaviours as developmental, not as summative judgments. This requires careful facilitation that frames the BMS as a guide for growth and improvement and not a rigid feedback rubric.
*Clarity of expectations: Trusting the choreography*	Trust is enhanced when the BMS provides consistent and visible categories, observable anchors, and a shared vocabulary for making the basis of feedback transparent.	Trust is undermined if the BMS, or some elements of it, feel confusing, irrelevant to clinical practice or inconsistently applied, causing learners to doubt both the tool and those using it.	Facilitators should invite learners to co-create the BMS that will be used in their simulation sessions. Introducing a BMS prior to use, accompanied by clear rationale and training, can bolster trust by demonstrating alignment with professional standards.
*Identity work: The dancer in the mirror*	BMS-based feedback can help learners see themselves as competent, align their behaviours with professional expectations, and feel pride in their growth and development.	If feedback is based disproportionally on negative behavioural descriptors, it risks undermining learners’ fragile professional identities, leaving them with self-doubt.	Facilitators should be cognisant of balancing affirmations with developmental suggestions, ensuring that positive aspects are emphasised even when significant scope for improvement is identified. Facilitators should also be attuned to using BMS to highlight identity growth alongside performance gaps.
*Peer critique: Dancing in sync*	Shared language, a structured framework and explicit exemplars can legitimise peer-to-peer observations through an agreed professional lens, reducing social discomfort.	Peer critique remains socially charged and uncomfortable, and use of BMS may exacerbate a sense of peer comparison.	BMS should be introduced as a framework to enable constructive peer feedback. Assisting learners to use BMS with an emphasis on descriptive rather than evaluative language can enhance peer feedback quality and reduce interpersonal tension.
*Agency and ownership: Leading the dance*	BMS can promote agency by making feedback specific and actionable, supporting learner agency in setting goals and planning change.	If learners perceive the system as externally imposed, they may disengage, feeling that they don’t have ownership of the strategies for improvement.	Learners and facilitators should co-interpret behavioural observations in debriefings, encouraging ownership and collaborative goal setting. This works well within an established debriefing model that allows space for learner reflections and discussion.

*BMS* Behavioural marker system

Although presented in both the text and Table 3 as discrete for clarity, these five themes were deeply interrelated. Emotional vulnerability often intersected with peer critique, where the risks of embarrassment or humiliation constrained the quality of feedback. Perceptions of trust and credibility were closely tied to learners’ sense of identity: feedback felt affirming when delivered transparently and constructively but threatening when it undermined professional self-concept. Similarly, the extent to which learners exercised agency in acting on feedback depended not only on their individual feedback literacy but also on the relational conditions of the educational alliance. The use of BMS shaped all of these dynamics, at times reducing vulnerability, building trust, and legitimising peer feedback, but at other times amplifying risks through inconsistency or overly rigid framing. Much like a dance, the socio-emotional processes of simulation feedback unfolded as interdependent movements, with each theme influencing and being influenced by the others rather than existing alone.

## Discussion

This study extends understanding of behavioural marker systems by illuminating their socio-emotional effects within simulation debriefing. While BMS have been promoted primarily as tools to enhance feedback consistency and transparency, our findings show that their influence goes beyond technical structuring. By shaping how learners experienced vulnerability, trust, identity, peer critique, and agency, BMS actively mediated the relational conditions under which feedback during debriefing was offered and taken up. This moves the literature beyond viewing BMS simply as neutral scaffolds for observation, highlighting their role as dynamic social instruments that can both enable and constrain learning.

Our findings highlight a striking tension in peer critique - trainee pharmacists were often reluctant to deliver negative feedback to colleagues, framing positivity as a way of preserving social harmony. Yet the same trainees expressed a strong desire to receive constructive critique themselves, recognising it as essential for growth and improvement. This mismatch, echoed in the higher education literature [29, 30], risks undermining learning, as what is intended as kindness may, in effect, be a disservice by denying peers actionable insights. BMS may offer a way to resolve this tension by legitimising critique. Prior research suggests that BMS provide a shared vocabulary and observable anchors that depersonalise judgment and reduce the perception of subjectivity [13, 14]. The provision of structured tools for peer feedback has been shown to enhance feedback quality [31]. Our study adds to this by suggesting that BMS can empower learners to deliver the feedback they would want to receive, reframing criticism as an objective observation against agreed behavioural standards, rather than a personal attack. In this sense, BMS can shift peer critique from a socially risky act to a professional responsibility. This aligns with literature on feedback cultures in health professions education, where explicit structures and legitimising frameworks have been shown to enable more candid, constructive exchanges among learners [9]. Thus, BMS may not only enhance the transparency of facilitator feedback but also recalibrate the dynamics of peer feedback, supporting a more reciprocal and effective exchange.

Trust emerged in our findings as a fragile but essential condition for effective feedback. While the structure and transparency of a BMS could enhance trust by making the basis of judgments explicit, any perception that the tool lacked credibility risked broader consequences. Trainees questioned categories that felt inauthentic to practice, resonating with findings in the HPE literature that clinical relevance is a key factor in the perceived credibility of an assessment tool [32]. Trainees also noted inconsistency in how the tool was applied, and these doubts eroded not only confidence in the BMS itself but also in the facilitator. This finding underscores the relational stakes of tool use: the credibility of the system and the credibility of the educator are intertwined. Prior work has shown that learners judge the trustworthiness of feedback sources before deciding whether to engage [33], and our study suggests that BMS legitimacy is part of this evaluative calculus. These insights highlight the importance of rigorous validation studies for BMS in health professions education. Without clear evidence that the categories reflect authentic professional practice, tools risk being seen as artificial and learners disengage. Conversely, well-validated BMS can act as credible scaffolds that strengthen both the educational alliance and learners’ willingness to internalise and act on feedback [34].

### Strengths and limitations

A strength of this study lies in its strong contextual grounding, involving a national cohort of trainee pharmacists participating in a bespoke simulation program, using a BMS specifically developed and validated for their profession. The use of established theoretical frameworks (feedback literacy and educational alliance) alongside a systematic analytic approach enhanced both rigour and interpretive depth, while reflexivity within a multidisciplinary research team helped to surface and question assumptions. However, limitations include the volunteer nature of participation, which may have led to self-selection distortion, and the single professional group studied, which limits transferability to other health professions or settings. Consistent with recent guidance on discussing transferability in qualitative health professions education [35], we have sought to provide sufficient contextual detail about the participants, simulation design and local training structures to enable readers to judge the applicability of these findings to their own settings. The research team’s prior involvement in the development of the PhaBS tool may have introduced unrecognised conflicts of interest, despite our efforts to reflect critically on positionality. The team’s methodological choices were informed by the concept of information power, and these decisions both enabled and constrained what was seen in the data. A relatively narrow study aim and highly specific sample of trainee pharmacists and pharmacist facilitators, combined with strong theoretical grounding in feedback literacy and the educational alliance, supported a decision to work with a modest sample. At the same time, the research team recognise that this design limits transferability to other professions, simulation modalities and institutional cultures where BMS may be used differently.

### Further work

Future research could focus on establishing whether training sessions for simulation participants can improve engagement with BMS, and the quality of peer feedback. Building on the theme of agency and ownership, future studies could also examine the effects of learner co-construction of BMS on the learners’ abilities to use such tools, and their trust in the tool’s validity. In addition, work could examine how a BMS shapes feedback literacy and the educational alliance when engaging with others involved in debriefing (for example, co-facilitators, supporting staff or simulated patients), and how this in turn influences the emotional and relational texture of the debriefing.

## Conclusions

This study shows that BMS are not simply neutral scaffolds for feedback, but dynamic social instruments that shape the relational ‘dance’ of debriefing. By influencing how learners experienced emotional vulnerability, trust, identity, peer critique, and agency, the BMS mediated key dimensions of both feedback literacy and the educational alliance. At their best, they provided choreography that depersonalised critique, legitimised peer feedback, affirmed professional becoming, and supported learners in taking the lead in their own development. Yet if applied rigidly or inconsistently, they risked missteps that undermined trust and damaged fragile identities. In this way, BMS actively shape the socio-emotional dynamics of debriefing.

## Supplementary Information

Supplementary Material 1.

## Data Availability

The datasets generated and/or analysed during the current study are not publicly available as consent for sharing of their data with other researchers was not obtained from study participants.

## Abbreviations

SBE: Simulation-based education
BMS: Behavioural marker system
PhaBS: Pharmacists’ Behavioural Skills
NOTSS: Non-technical Skills for Surgeons
ANTS: Anaesthetists’ Non-Technical Skills
Medi-StuNTS: Medical Students’ Non-Technical Skills
