# Supplementary material for: The delicate dance of debriefing: exploring how behavioural marker systems influence the socio-emotional dynamics of simulation practice
Source: Adv Simul (Lond). 2026 Feb 6;11:20. doi: 10.1186/s41077-026-00411-4 (PMC12973708; doi:10.1186/s41077-026-00411-4)
Supplement: Supplementary file 1 — Supplementary Material 1. [file 41077_2026_411_MOESM1_ESM.docx]

**Appendix 1: Semi-Structured Interview Guide**

*How does use of a behavioural marker system (PhaBS) influence the emotional and social aspects of debriefing, for both learners and facilitators?*

**Warm-up**

1. Can you tell me about your experience of the recent simulation sessions?

- What stood out to you most?
- How did you feel during the debriefing?

**Section 1: Emotional experiences and vulnerability (Feedback literacy: managing affect)**

1. Can you describe a moment in the debriefing where you felt particularly supported or encouraged?
2. Can you describe a moment that felt uncomfortable, exposing, or challenging?
3. How, if at all, did the PhaBS tool affect those feelings?

*Follow-up prompts:*

- Did it make feedback feel more personal or less personal?
- Did it make you feel more or less anxious?
- Did it affect how safe you felt to contribute?

**Section 2: Clarity, trust and credibility (Educational alliance: credibility and caring)**

1. How did having the PhaBS tool shape your sense of how fair, clear, or credible the feedback was?
2. Did you trust the feedback more, less, or about the same when the tool was used?

**Section 3: Identity and self-concept (Feedback literacy: appreciating feedback; Educational alliance: relational caring)**

1. How did the feedback you received using PhaBS influence how you saw yourself as a developing professional?

*Follow-up prompts:*

- Were there times when the feedback strengthened your confidence?
- Were there times when it made you doubt yourself?

**Section 4: Peer dynamics and collaboration (Educational alliance: collaboration; Feedback literacy: evaluative judgement)**

1. What was it like giving or receiving feedback from peers in the debriefing?
2. Did using the PhaBS tool change how easy or difficult it felt to give feedback to a peer?
3. Did the tool change how you received peer feedback?

**Section 5: Agency and use of feedback (Feedback literacy: acting on feedback)**

1. Did the PhaBS tool affect whether you felt you could act on the feedback you received?
2. Did it alter whether you felt you had ownership of the feedback or your next steps for improvement?

**Closing**

1. Is there anything I haven’t asked about that feels important to your experience?
